# Peak alpha frequency and alpha power spectral density as vulnerability markers of cognitive impairment in Parkinson’s disease: an exploratory EEG study

**DOI:** 10.3389/fnins.2025.1575815

**Published:** 2025-04-29

**Authors:** Yuqing Zhao, Jiayu Cai, Jian Song, Haoran Shi, Weicheng Kong, Xinlei Li, Wei Wei, Xiehua Xue

**Affiliations:** 1 The Affiliated Rehabilitation Hospital, Fujian University of Traditional Chinese Medicine, Fuzhou, China; 2 College of Rehabilitation Medicine, Fujian University of Traditional Chinese Medicine, Fuzhou, China; 3 Fujian Provincial Rehabilitation Industrial Institution, Fujian Provincial Key Laboratory of Rehabilitation Technology, Fujian Key Laboratory of Cognitive Rehabilitation, Fuzhou, China

**Keywords:** cognitive impairment, peak alpha frequency, power spectral density, Parkinson’s disease, EEG

## Abstract

**Background:**

Cognitive impairment substantially impacts quality of life in Parkinson’s disease (PD), yet current biomarker frameworks lack sensitivity for detecting early-stage cognitive decline. While peak alpha frequency (PAF) and alpha power spectral density (PSD) have emerged as potential electrophysiological markers, prior studies primarily focused on global cortical measures, neglecting region-specific variations that may better reflect the heterogeneous nature of PD-related cognitive impairment (PDCOG). To address this gap, we conducted the first multiregional comparative analysis of PAF and alpha PSD between PDCOG and PD with normal cognition patients (PDNC).

**Methods:**

Data from 76 participants (44 PD, 32 healthy controls) at The Affiliated Rehabilitation Hospital of Fujian University of Traditional Chinese Medicine (March–July 2024) were analyzed. PAF and alpha PSD were computed across brain regions; cognitive function was assessed via MoCA.

**Results:**

Global PAF was reduced in PD vs. controls (*p* < 0.05) and correlated with cognition. PDCOG showed lower alpha PSD in parieto-occipital/posterior temporal regions (P3, P4, O1, T5, T6, PZ) vs. PDNC (*p* < 0.05), with these regions correlating with MoCA scores. ROC analysis identified P3, PZ, and T6 alpha PSD as optimal discriminators (AUC: 0.77–0.758). Executive function inversely correlated with alpha PSD in right posterior temporal/left occipital regions.

**Conclusion:**

PAF differentiates PD from controls and links to global cognition, while regional alpha PSD (notably P3, PZ, T6) effectively distinguishes PDCOG from PDNC. These findings underscore regional QEEG’s utility in PD cognitive assessment, though sensitivity limitations warrant optimization.

## 1 Introduction

Parkinson’s disease (PD), a multisystem neurodegenerative disorder, is defined by both motor deficits (Barone et al., 2009) and heterogeneous nonmotor symptoms, including cognitive decline (Chaudhuri et al., 2006). Individuals with PD generally exhibit a heightened susceptibility to dementia compared to the general population, with PD-dementia (PDD) incidence reaching as high as 46% in PD patients with a history exceeding 10 years (Williams-Gray et al., 2013). PD patients with cognitive impairment (PDCOG) may experience deficits across multiple cognitive domains (Harvey, 2019). These deficits profoundly impair quality of life (Chandler et al., 2021), incur significant socioeconomic burdens, and predict faster disease progression—even surpassing motor symptoms in early-stage impact. Current cognitive scales (e.g., MoCA) lack neurobiological specificity, failing to link deficits to underlying pathology [e.g., alpha rhythm dysregulation (Saredakis et al., 2019)]—a gap hindering precision care.

Quantitative electroencephalography (QEEG) is a renowned, non-invasive, and cost-effective technique for capturing the electrical activity of the brain. It offers quantitative insights into brain functions, including peak alpha frequency (PAF) and power spectral density (PSD). This method has gained notable attention in recent years due to its excellent spatial resolution in detecting neuronal electrical activity (Babiloni et al., 2020; Novak et al., 2021; Geraedts et al., 2018). Cognitive decline is associated with specific neurodegenerative patterns, such as corticostriatal pathway dysfunction and alpha rhythm dysregulation (Zhou et al., 2024). For instance, decreased occipital alpha/theta ratios are predictive of visuospatial deficits (Jaramillo-Jimenez et al., 2021), whereas parietal alpha PSD is correlated with overall cognitive function (MoCA scores) (Anjum et al., 2024). By distinguishing between PDCOG and PDNC, clinicians can identify patients at risk of rapid progression or PDD early on, thereby facilitating biomarker-driven monitoring.

PD patients exhibit globally reduced PAF, suggesting dopaminergic dysfunction (Kamei et al., 2010; Morita et al., 2011; Caviness et al., 2015; Aarsland et al., 2021), while regional alpha PSD reductions in parieto-occipital regions specifically mark PDCOG (Jaramillo-Jimenez et al., 2021). These patterns align with cognitive domain vulnerabilities (Rea et al., 2021; Polverino et al., 2022; Yılmaz et al., 2020): low parietal alpha PSD predicts executive dysfunction, whereas posterior temporal declines associate with memory deficits. Such biomarkers bridge the gap between symptom-based scales and pathophysiology, offering tools for subtyping and targeted interventions.

Based on evidence suggesting that alpha oscillations are fundamental to cognitive control networks, previous studies have not sufficiently explored the role of PAF and alpha PSD in differentiating cognitive impairment levels in PD, we are exploring whether PAF and alpha PSD can objectively differentiate between PDCOG and PDNC. Through the association of regional QEEG signatures with cognitive profiles, our goal is to overcome the restrictions of present evaluations and move towards a pathology-informed approach for PD phenotyping.

## 2 Materials and methods

### 2.1 Participants and cognitive measures

A cross-sectional study design was employed in this research. From March to July 2024, this study recruited 44 participants with Parkinson’s disease (24 females and 20 males) from the Rehabilitation Hospital affiliated with Fujian University of Traditional Chinese Medicine. Additionally, 32 healthy controls (HC) (20 females and 12 males), matched by age and gender were recruited. All subjects were fully informed and signed informed consent. The study was approved by the Ethics Committee of the Rehabilitation Hospital affiliated with Fujian University of Chinese Medicine (No. 2023KY-056-002).

According to both the United Kingdom Parkinson disease (UKPD) Society Brain Bank criteria (Gibb and Lees, 1988) clinical diagnostic criteria for Parkinson’s disease (PD) (MDS-PD criteria) (Gill et al., 2008), a total of 44 PD patients were recruited from the Rehabilitation Hospital affiliated with Fujian University of Chinese Medicine (Fuzhou, China). All the subjects were native Chinese speakers and right-handed. The inclusion criteria of the healthy control group were: (1) aged between 45 and 80 years; (2) The age and gender were matched with those in PD group; and (3) No history of neurological or mental illness.

We used MoCA to quantify cognitive condition among participants as it is more sensitive to cognitive deterioration in PD (Gill et al., 2008; Vásquez et al., 2019; Chou et al., 2010). We defined cognitive impairment (PDCOG) as MoCA scores < 26 scores and cognitive normal (PDNC) as MoCA scores (Nasreddine et al., 2005; Dalrymple-Alford et al., 2010; Chou et al., 2014). All subjects completed the MoCA scale and EEG examination within 3 days after enrollment. As reported by Lam et al. (2013), we have redefined the five cognitive domains associated with each MoCA score (Memory, Visuospatial, Language, Attention Executive). Clinical and demographic characteristics of enrolled PD and HC subjects are reported in Table 1 and Supplementary Table 1.

**Table 1:** Clinical characteristics of PD and HC.

Item	PD (*n* = 44)			HC (*n* = 32)	t/*x*^2^	*p*
	Total (*n* = 44)	PDCOG (*n* = 31)	PDNC (*n* = 13)			
Gender (Male/Female)	20/24	17/14	3/10	12/20	−0.69	0.495
Age (year)	66.25 ± 7.70	67.45 ± 5.71	63.38 ± 10.87	65.78 ± 8.96	0.25	0.807
Education level (year)	10.48 ± 4.67	9.74 ± 4.64	12.62 ± 3.38	11.97 ± 3.29	−1.63	0.126
Duration of disease (year)	4.34 ± 3.03	4.44 ± 3.19	4.12 ± 2.72	–	–	–
HY stage	2.32 ± 0.64	2.39 ± 0.67	2.15 ± 0.56	–	–	–
MoCA score	22.77 ± 4.50	20.65 ± 3.48	27.85 ± 1.63	25.63 ± 1.43	−3.94	**<0.001** ^*^

*t*-test: Compared to PD bold value means *p < 0.05. While categorical variable is presented with number of patients. HC, healthy controls; PDCOG, PD with cognitive impairment; PDNC, PD with normal cognition; MoCa, Montreal Cognitive Assessment; HY, Hoehn and Yahr stage; MDS-UPDRSIII, the part III of the Movement Disorder Society-Sponsored Revision of the Unified Parkinson’s Disease Rating Scale; *p* value: difference between HCs and all PD groups.

### 2.2 PAF and alpha PSD recordings and preprocessing

#### 2.2.1 EEG acquisition process

In this study, three minutes of resting-state EEG activity were collected using the Cognitive and Autonomous Nervous Function Mapping EEG Monitor (NVX52 EEG Acquisition System, Nanjing NeuroMed Technology Group Co., Ltd., China). Nineteen standard EEG electrodes were placed on the scalp with an adjustable cap according to the internationally recognized 10–20 system, and an AA electrode was used as the reference (We use 2 electrodes, A1 and A2. AA = (A1 + A2) /2). During data collection, subjects were instructed to maintain a comfortable posture and were guided to close their eyes. The contact impedance between the electrodes and the scalp was strictly maintained below 20 KΩ (Lee et al., 2013).

#### 2.2.2 PAF and alpha PSD analyses

The recorded EEG data were subjected to comprehensive spectrum PSD analysis, encompassing all 19 channels. The sampling rate used in the data acquisition process is 500 Hz. A broad band power spectrum (0.5–48 Hz) was obtained through Fast Fourier transformation of the time-series, from which absolute and relative spectral power were computed for six frequency bands (delta (0.5–4 Hz), theta (4–8 Hz), alpha (8–13 Hz), beta (13–30 Hz) and gamma (30–48 Hz)). For FFT calculation we use “Hann window function” with “Window length = 4 s with 50% overlapping.” And for “Window length = 4 s” the resolution of frequency about 0.25 Hz. Given our focus on alpha band, this study exclusively analyses the alpha band (Schleiger et al., 2014). The PAF was identified as the frequency point exhibiting the highest PSD within the alpha band, ranging from 8 to 13 Hz.

The quality of the collected EEG data were manually inspected and preprocessed in EEGLAB. The Infomax Independent Component Analysis (ICA) module was used to decompose the EEG data and remove artifact components, including ocular and muscle artifacts (Delorme et al., 2007; Pion-Tonachini et al., 2019). The study focused on the frequency-power spectrum, particularly the peak frequency of the alpha wave, which was defined as the frequency point with the highest PSD within the alpha band, covering all 19 leads.

### 2.3 Statistical analyses

All analyses were performed using IBM SPSS Statistics (version 26.0) with a two-tailed significance threshold of *p* < 0.05. Continuous variables were compared between groups using independent samples *t*-tests for normally distributed data (assessed via Shapiro–Wilk test) and Mann–Whitney U tests for non-normally distributed datasets. To identify predictor variables of cognitive outcomes, multiple linear regression models were constructed, incorporating peak alpha frequency (PAF) and alpha band power spectral density (PSD) as independent variables, with the MoCA total score and its subdomains serving as dependent variables.

The diagnostic utility of PAF and alpha PSD in predicting Parkinson’s disease-related cognitive impairment (PDCOG) was evaluated through receiver operating characteristic (ROC) curve analysis, with sensitivity and specificity quantified by the area under the curve (AUC). To address multiple comparisons in correlation analyses, associations between PAF, alpha PSD, and cognitive scale scores were examined using Pearson’s correlation with false discovery rate (FDR) correction; significant correlations were defined by both raw *p* < 0.05 and FDR-adjusted *q* < 0.05, and the control error discovery rate was 5%.

## 3 Results

### 3.1 The results of demographic data and clinical assessment

This study included patients with PD (*n* = 44) and HC (*n* = 32). There was no difference in gender, age and education level between the two groups (*p* > 0.05). The MoCA score of the PD group was lower than that of the HC (*p* < 0.05), see Table 1.

Furthermore, a comparative analysis was conducted on demographic and clinical assessment data between PD with cognitive impairment (PDCOG, *n* = 31) and those normal cognition (PDNC, *n* = 13). There were no significant differences in gender, age, duration of disease, Hoehn-Yahr stage between the two groups (*p* > 0.05). However, the PDCOG group had significantly lower educational level and MoCA scores compared to the PDNC group (*p* < 0.05), see Table 1 and Supplementary Table 1.

### 3.2 Comparison of the peak alpha frequency between PD and HC

**Figure 1:**
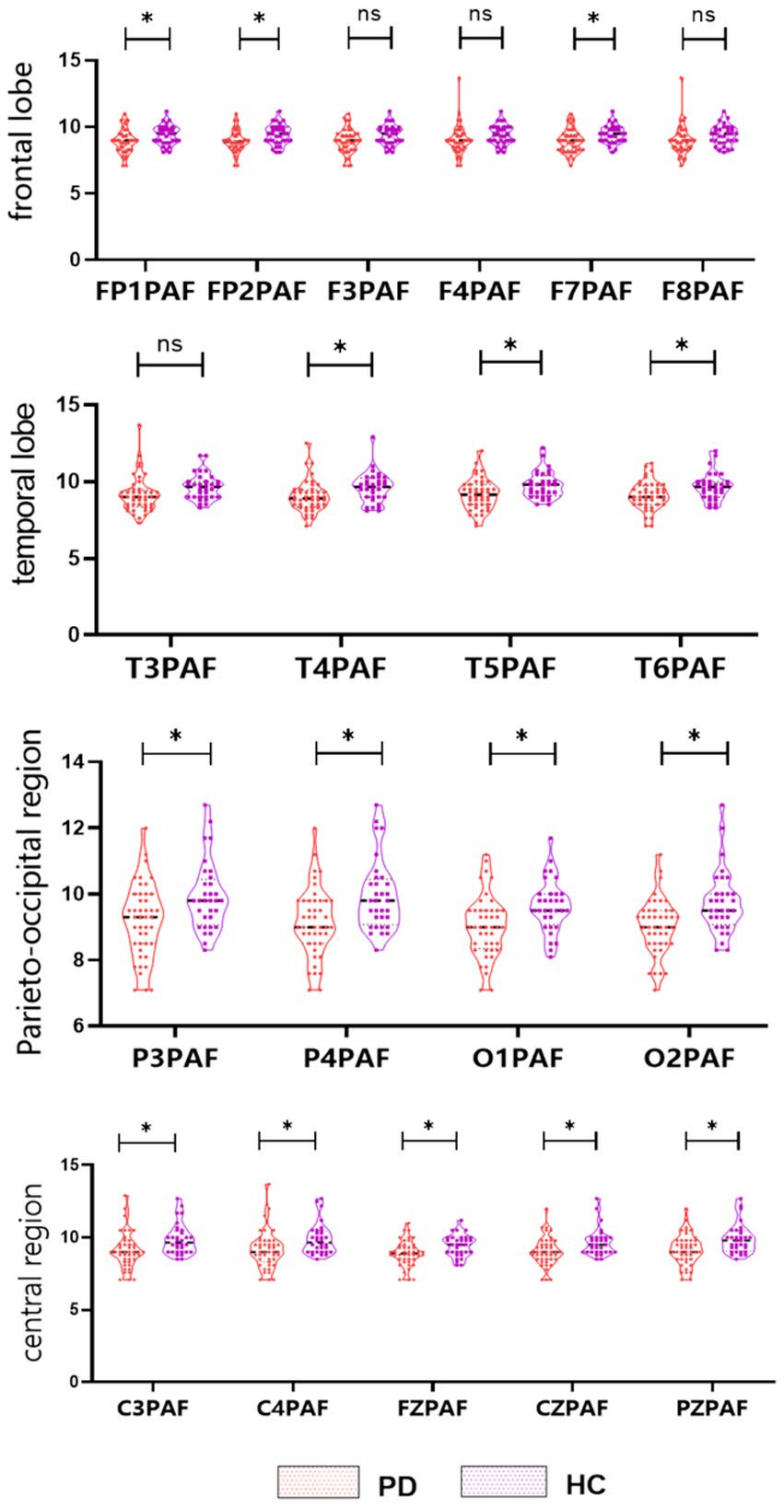
Violin plot of the PAF for PD and HC. The PAF in the bilateral frontal (FP1, FP2, F7), temporal (T4, T5, T6), parietal-occipital (P3, P4, O1, O2) and central (C3, C4, FZ, CZ, PZ) regions were significantly lower in PD than in the HC (*p* < 0.05). PAF, peak alpha frequency. * *p* < 0.05, ** *p* < 0.01, *** *p* < 0.001.

The results demonstrated that the PAF in the PD group was significantly lower than that in the HC group (*p* < 0.05). This reduction was observable in multiple brain areas, specifically the frontal region (FP1, FP2, F7) (*p* < 0.05), temporal region (T4, T5, T6) (*p* < 0.05), central region (C3, C4, FZ, CZ, PZ) (*p* < 0.05), and parietal-occipital region (P3, P4, O1, O2) (*p* < 0.05), see Figure 1 and Supplementary Table 2.

### 3.3 Comparison of the alpha PSD between PDCOG and PDNC

**Figure 2:**
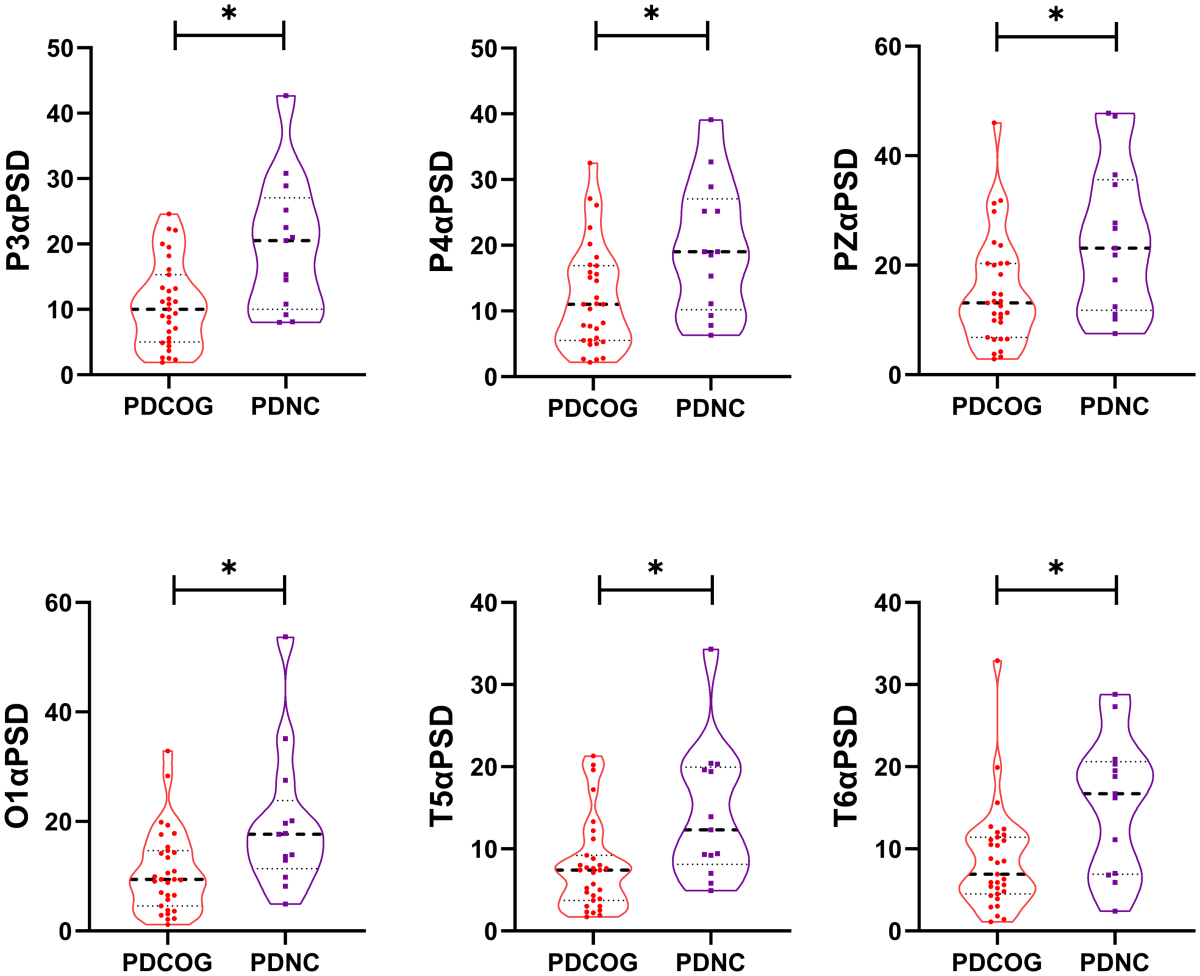
Violin plot of peak alpha PSD for PDCOG and PDNC. The PSD in the parieto-occipital region (P3, P4, PZ, O1), temporal (T3, T4, T5, T6), parietal-occipital (P3, P4, O1, O2) and the temporal region (T5, T6) regions were significantly lower in PDCOG than in the PDNC (*p* < 0.05). PSD, power spectral density. * *p* < 0.05.

After the Mann–Whitney U test, significant differences were observed in P3α PSD (*p* = 0.019), P4α PSD (*p* = 0.030), PZα PSD (*p* = 0.035), O1α PSD (*p* = 0.030), T5α PSD (*p* = 0.025) and T6α PSD (*p* = 0.025) between the PDCOG group and the PDNC group, while no differences were found in other regions (*P*>0.05), see Figure 2 and Supplementary Table 3.

### 3.4 Correlation analysis between PAF and MoCA total score and subitems scores in PD group

The correlation analysis conducted in this study revealed notable negative correlations between MoCA scores and PAF values in the temporal–parietal region (T5, P4, PZ) as well as the midline region (CZ). Specifically, the correlation coefficients and corresponding *p*-values were as follows: (*r* = −0.321, *p* = 0.034), (*r* = −0.344, *p* = 0.022), (*r* = −0.345, *p* = 0.022), and (*r* = −0.336, *p* = 0.026), the results survived FDR correction (*q* = 0.034).

There were also significant negative correlations between the temporal–parietal region PAF (T5, P4, PZ) with visuospatial scores. The correlation coefficients and *p*-values were (*r* = −0.344, *p* = 0.022), (*r* = 0.361, *p* = 0.016) and (*r* = −0.35, *p* = 0.02), respectively, the results survived FDR correction (*q* = 0.022).

**Figure 3:**
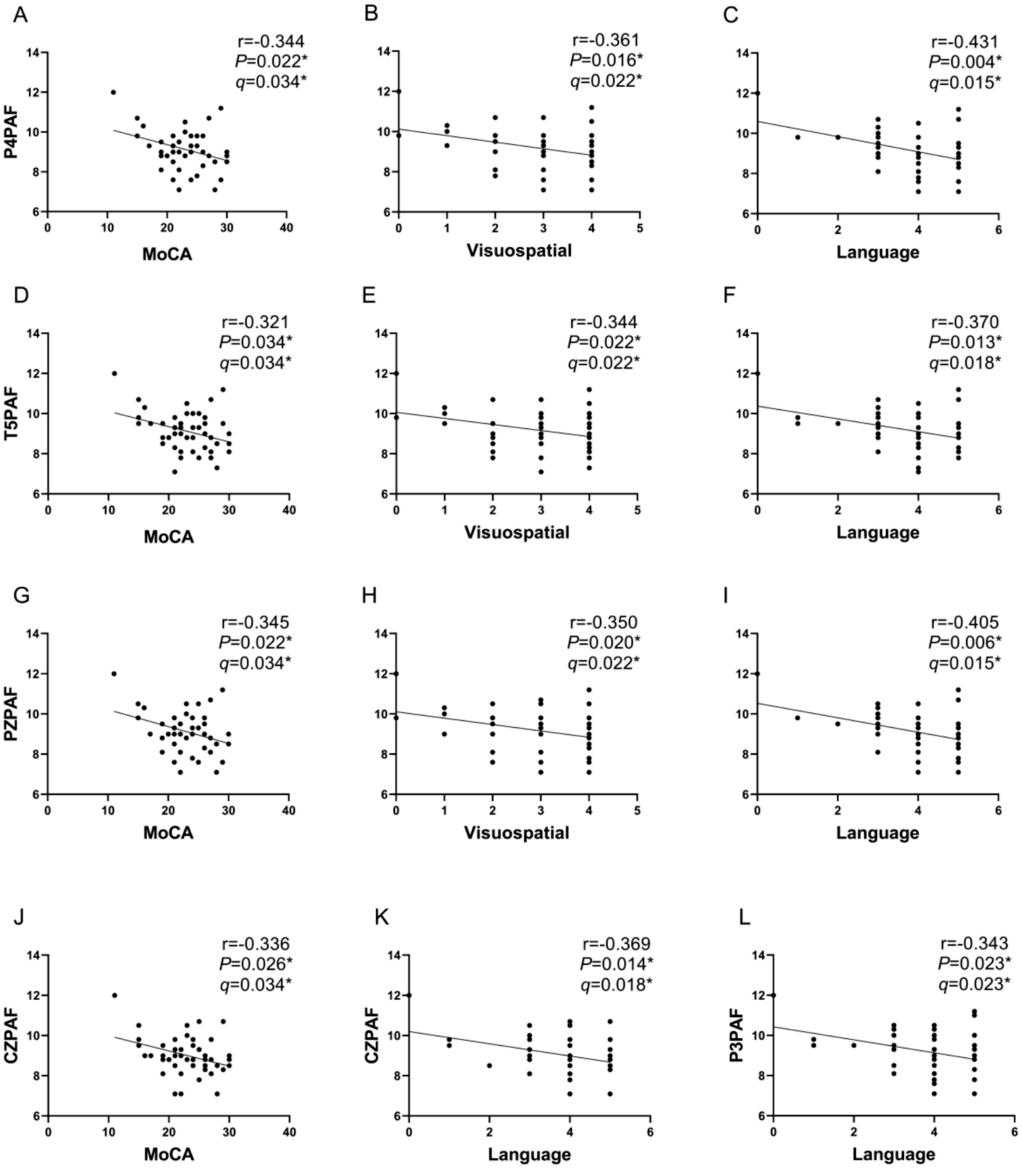
Correlation analysis between PAF and MoCA and subitems scores in PD group. **(A–C)**: A significant negative correlation was found between P4PAF and MoCA scores **(A)**, visuospatial scores **(B)**, language scores **(C)** in patients with PD. **(D–F)**: A significant negative correlation was found between T5PAF and MoCA scores **(E)**, visuospatial scores **(F)**, language scores **(G)** in patients with PD. **(G–I)**: A significant negative correlation was found between PZPAF and MoCA scores **(G)**, visuospatial scores **(H)**, language scores **(I)** in patients with PD. **(J)**: A significant negative correlation was found between CZPAF and MoCA scores in patients with PD. **(K)**: A significant negative correlation was found between CZPAF and language scores in patients with PD. **(L)**: A significant negative correlation was found between P3PAF and language scores in patients with PD. *q*: FDR corrected *p* value with Benjamini-Hochberg. *q*<0.05*.

Additionally, temporal–parietal region PAF (T5, P3, P4, PZ) and midline region PAF (CZ) showed significant negative correlations with language scores. The correlation coefficients and *p*-values were T5 (*r* = −0.37, *p* = 0.013), P3 (*r* = −0.343, *p* = 0.023), P4 (*r* = −0.431, *p* = 0.004), PZ (*r* = −0.405, *p* = 0.006) and CZ (*r* = −0.369, *p* = 0.014), respectively, the results survived FDR correction (*q* = 0.018, *q* = 0.023, *q* = 0.015, *q* = 0.015, *q* = 0.018), see Figure 3.

### 3.5 Correlation analysis between PSD and MoCA subitems scores

The correlation analysis revealed that in the PDCOG group, alpha PSD in temporal–parietal-occipital region (P4, O1, T6, PZ) were negatively correlated with executive function scores (*p* < 0.05). The correlation coefficients and *p*-values were P4 (*r* = 0.363, *p* = 0.045), O1 (*r* = 0.384, *p* = 0.033), T6 (*r* = 0.402, *p* = 0.025) and PZ (*r* = 0.366, *p* = 0.043), respectively, the results survived FDR correction (*q* = 0.045).

**Figure 4:**
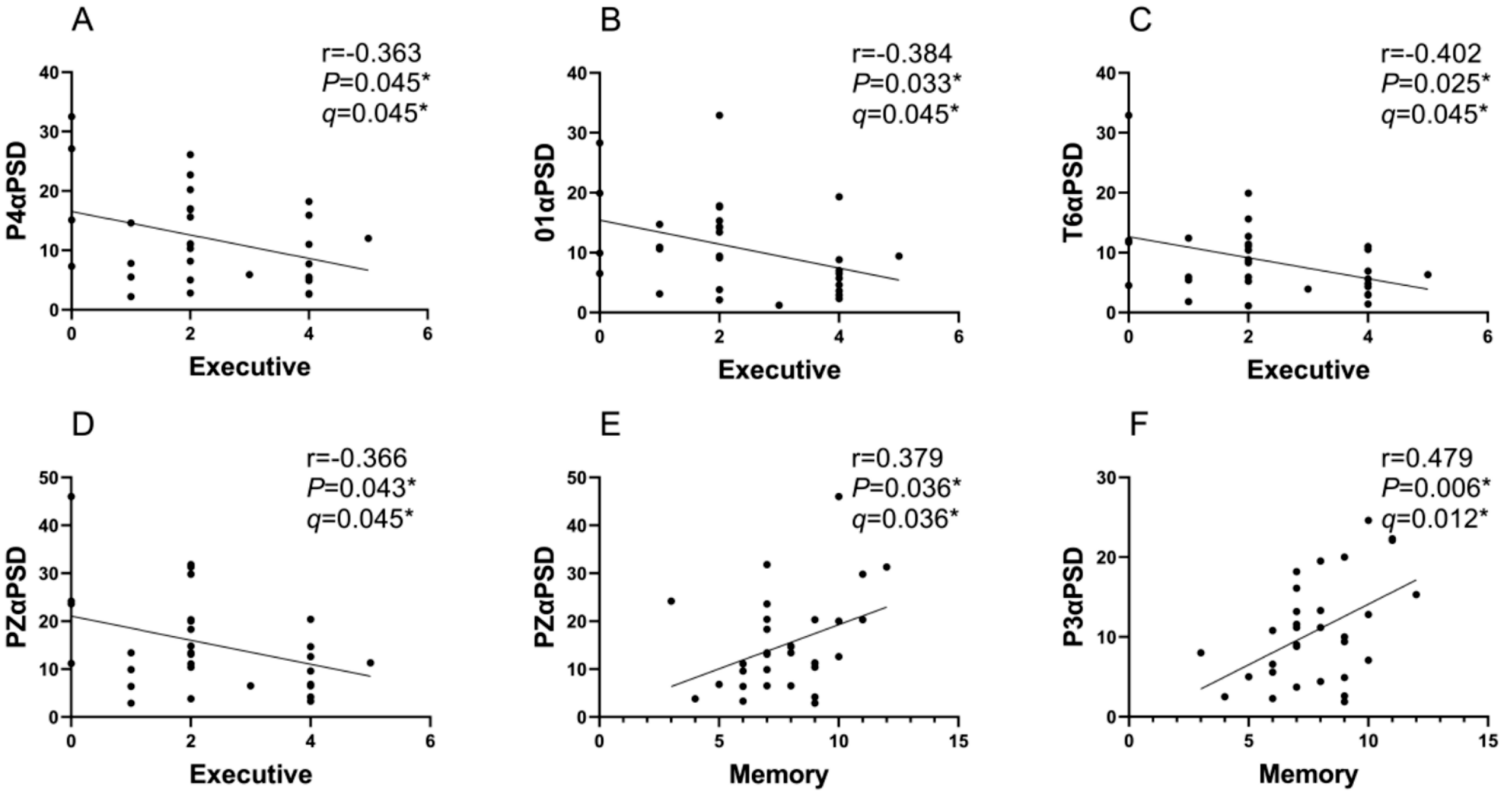
Correlation analysis between the peak alpha PSD and MoCA and subitems scores in PDCOG group. **(A–D)**: A significant negative correlation was found between P4*α*PSD **(A)**, O1αPSD **(B)**, T6αPSD **(C)**, PZαPSD **(D)** and executive scores in patients with PDCOG. **(E)**: A significant positive correlation was found between PZαPSD and memory scores in patients with PDCOG. **(F)**: A significant positive correlation was found between P3αPSD and memory scores in patients with PDCOG. *q*: FDR corrected *p* value with Benjamini-Hochberg. *q*<0.05*.

In contrast, alpha PSD in the parietal region (PZ, P3) showed a positive correlation with memory function (*p* < 0.05). The correlation coefficients and *p*-values were (*r* = 0.379, *p* = 0.036) and (*r* = 0.479, *p* = 0.006), respectively, the results survived FDR correction (*q* = 0.036, *q* = 0.012), see Figure 4.

### 3.6 ROC curves for PAF in the diagnosis of PD

**Figure 5:**
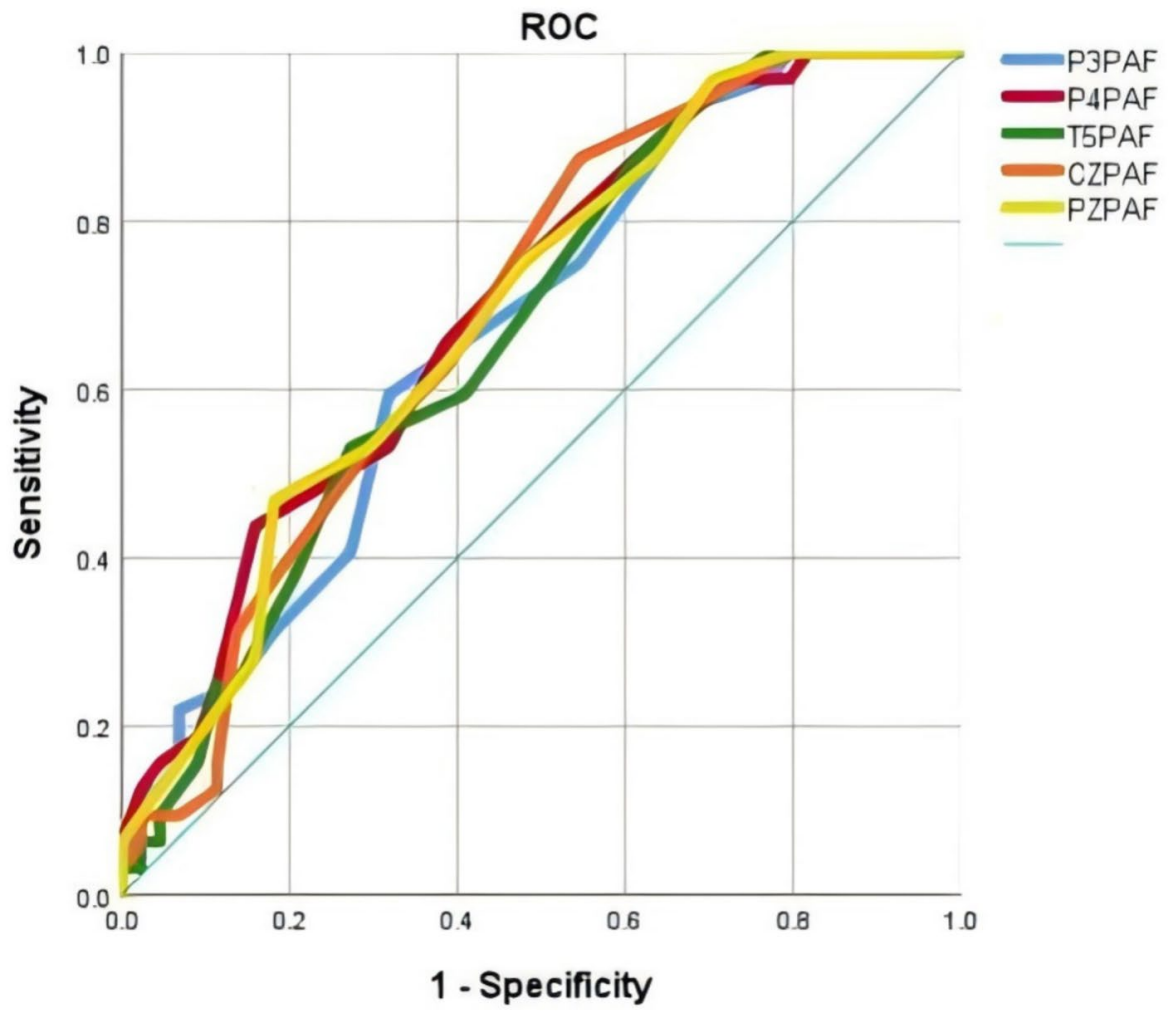
ROC curve analysis was used to measure the AUC of PAF. The AUC was 0.673 for P3PAF (blue curve), 0.701 for P4PAF (purple curve), 0.674 for T5PAF (green curve), 0.693 for CZPAF (yellow curve) and 0.694 for PZPAF (orange curve).

We conducted ROC curve analyses to investigate whether P3PAF, P4PAF, T5PAF, CZPAF, PZPAF might facilitate discrimination between PD patients and HC (see Figure 5). The areas under the curves (AUC) for P3PAF was 0.673, with a sensitivity of 59.4%, a specificity of 68.2%, and a cutoff of 9.65. The AUC for P4PAF was 0.701, with a sensitivity of 43.8%, a specificity of 84.1%, and a cutoff of 9.9. The AUC for T5PAF was 0.674, with a sensitivity of 87.5%, a specificity of 38.6%, and a cutoff of 8.9. The AUC for CZPAF was 0.693, with a sensitivity of 87.5%, a specificity of 45.5%, and a cutoff of 8.9. The AUC for PZPAF was 0.694, with a sensitivity of 46.9%, a specificity of 81.8%, and a cutoff of 9.9 (details in Table 2).

**Table 2:** ROC curve thresholds and corresponding TPR/FPR values for PAF.

Indices	AUC	Cut-off value	*p*	Sensitivity	Specificity	*95%CI*
P3PAF	0.673	9.65	0.01	0.594	0.682	(0.553, 0.793)
P4PAF	0.701	9.9	0.003	0.438	0.841	(0.585, 0.817)
T5PAF	0.674	8.9	0.01	0.875	0.386	(0.555, 0.794)
CZPAF	0.693	8.9	0.004	0.875	0.455	(0.576, 0.810)
PZPAF	0.694	9.9	0.004	0.469	0.818	(0.577, 0.811)

Detail data of ROC curves of PAF for the discrimination of PD and HC. AUC, areas under the curves.

### 3.7 ROC curves for alpha PSD indices in the diagnosis of PDCOG

**Figure 6:**
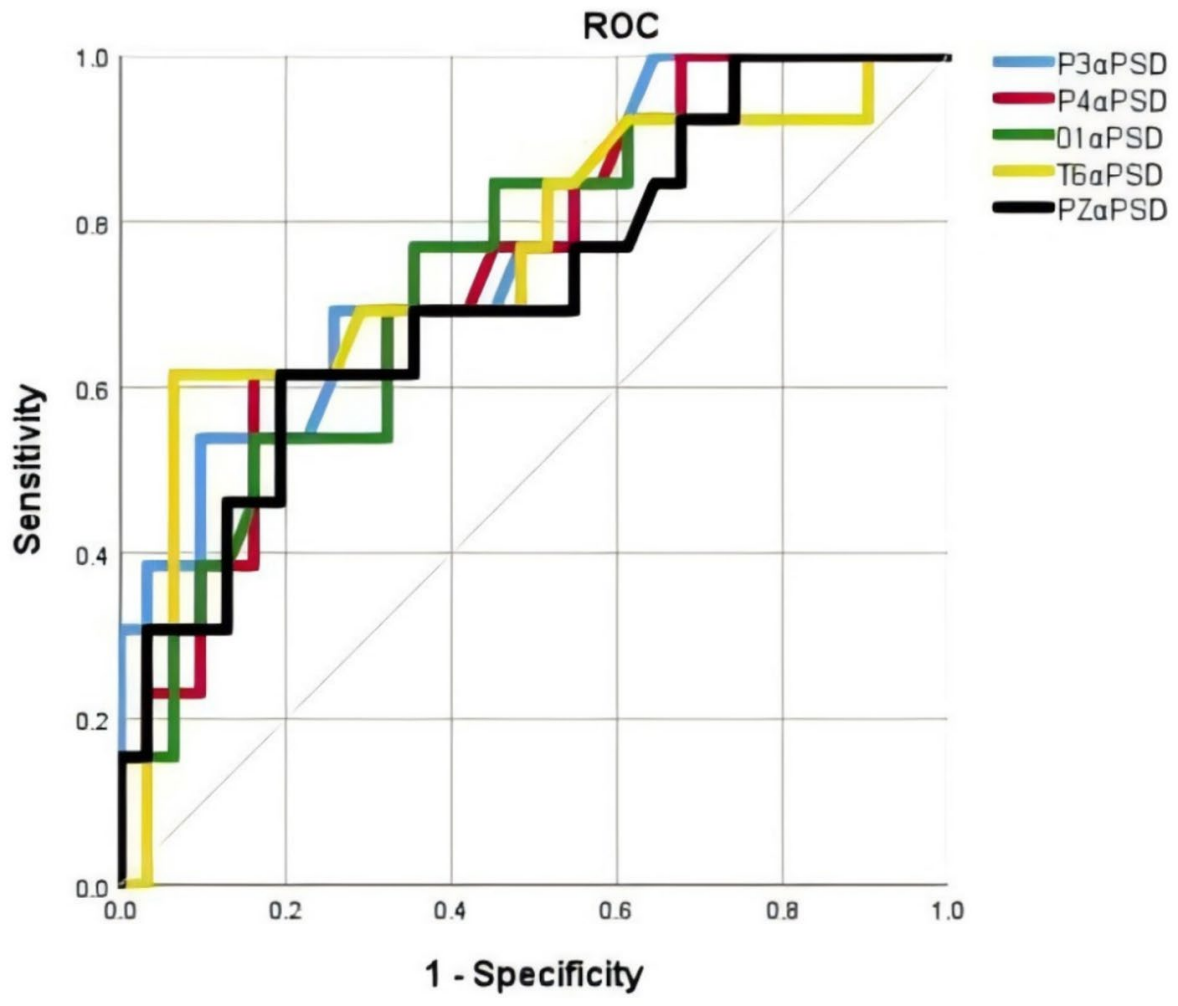
ROC curves for the evaluation of the utility of alpha PSD indices for the discrimination of PDCOG from PDNC. ROC curve analysis was used to measure the AUC of the alpha PSD indices. The AUC was 0.77 for P3 α PSD (blue curve), 0.747 for P4α PSD (red curve), 0.743 for O1α PSD (green curve), 0.758 for T6α PSD (yellow curve) and PZα PSD (black curve).

We conducted ROC curve analyses to investigate whether P3α PSD, P4α PSD, O1α PSD, T6α PSD and PZα PSD might facilitate discrimination between PDCOG patients and PDNC patients (Figure 6). The areas under the curves (AUC) for P3α PSD was 0.77, with a sensitivity of 53.8%, a specificity of 90.3%, and a cutoff of 20.25. The AUC for P4α PSD was 0.747, with a sensitivity of 61.5%, a specificity of 83.9%, and a cutoff of 18.35. The AUC for O1α PSD was 0.743, with a sensitivity of 76.9%, a specificity of 64.5%, and a cutoff of 11.9. The AUC for T6α PSD was 0.758, with a sensitivity of 61.5%, a specificity of 93.5%, and a cutoff of 15.9. The AUC for PZα PSD was 0.758, with a sensitivity of 61.5%, a specificity of 80.6%, and a cutoff of 9 (details in Table 3).

**Table 3:** ROC curve thresholds and corresponding TPR/FPR values for alpha PSD indices.

Indices	AUC	Cut-off value	*p*	Sensitivity%	Specificity%
P3α PSD	0.770	20.25	0.005	53.8	90.3
P4α PSD	0.747	18.35	0.01	61.5	83.9
O1α PSD	0.743	11.9	0.012	76.9	64.5
T6α PSD	0.758	15.9	0.007	61.5	93.5
PZα PSD	0.758	9	0.024	61.5	80.6

Detail data of ROC curves of alpha PSD indices for the discrimination of PDCOG and PDNC. AUC, areas under the curves.

## 4 Discussion

The inherent rhythms captured in resting QEEG data offer invaluable neurophysiological insights into human cognition (Dringenberg, 2000; Schreckenberger et al., 2004; Klimesch et al., 2007). In recent years, the assessment of cognitive function using PAF and alpha PSD has emerged as a prominent area of research, garnering significant attention. Numerous studies have established a positive correlation between alpha activity and cognitive function (Williams Roberson et al., 2022). PAF and PSD parameters not only demonstrate the ability to differentiate between PD patients and healthy individuals, but also show promise as biomarkers for identifying cognitive deficits in PD (Arnaldi et al., 2017; Chaturvedi et al., 2017; Waninger et al., 2020; Schumacher et al., 2020). However, the question remains regarding the optimal utilization of PAF and PSD’s discriminatory capabilities in various EEG regions, particularly in differentiating between healthy controls and PD subjects, as well as between cognitively normal and impaired PD subjects. Our study presents distinct findings on this complex and contentious issue.

### 4.1 Characteristics of PAF and PSD in PD patients

In our study, we examined the disparities in PAF between healthy individuals and those diagnosed with PD. Our findings uncovered a significant decrease in overall PAF among PD patients relative to HC. The PAF serves as a highly sensitive indicator of cognitive performance. Moreover, the PAF fluctuates in accordance with the level of cognition (Klimesch, 1997). PAF is commonly understood as the frequency demonstrating the peak PSD within the 8 to 13 Hz alpha band. This frequency is thought to correlate strongly with cognitive processes (Keitel et al., 2019; Ramsay et al., 2021; Finley et al., 2024). Research has shown that PD patients without dementia display a lower frequency of alpha spikes compared to HC (Ye et al., 2022). Our findings revealed a discernible difference in PAF between the PD and HC groups, moreover, this difference was significantly correlated with cognitive assessment outcomes. Physiologically, PAF not only indicates heightened brain arousal and vigilance, facilitating visual information processing in the parietal, temporal, and occipital cortical regions, but is also associated with attention and cognitive performance (Babiloni et al., 2022). Our findings revealed a negative correlation between the posterior temporal pole and superior parietal PAF, and MoCA scores in PD patients. This primarily reflects a negative association with visual–spatial abilities. Furthermore, the superior parietal PAF also shows a negative correlation with language scores. These observations suggest that heightened neural electrophysiological activity in specific brain areas may play a role in compensatory mechanisms for cognitive decline. In some neurodegenerative diseases, the brain may maintain its function through some compensatory mechanisms. For instance, when the function of certain brain regions is impaired, other brain regions may increase their activity to compensate for this loss. In our study, the reduction of PAF might be related to the excessive synchronization of activity in certain brain regions, which could be a compensatory response by the brain to maintain cognitive function. However, such compensatory mechanisms may not always be effective and might even have a negative impact on cognitive function in some cases. These findings align with previous research (Zhang et al., 2020). A study investigated the correlation between resting-state PAF, PSD, aging, and cognition, revealing a negative association between alpha power and processing speed, particularly prominent in the frontal region (Cesnaite et al., 2023). However, our results specifically highlight a negative association between PSD and cognitive performance at the occipital pole. Additionally, we observed a positive correlation between PAF and both right and left temporal regions, related to interference suppression during working memory tasks. While our study did not directly establish a link between PAF and memory, we did find a noteworthy positive correlation between PSD and memory performance in PD patients, which merits further investigation.

However, no such difference was observed when comparing PDCOG and PDNC. Hence, we hypothesize that dopamine may also regulate PAF in PD (Wacker, 2018), but further research is required to verify this.

### 4.2 Reduced parieto-occipital alpha PSD in PDCOG patients

To distinguish PDCOG patients from PDNC patients based on their cortical electrical activity, our study compared the brain networks of the two subject groups through the analysis of ICA. In PDCOG patients we found a reduction of the alpha component in the parietal and occipital region. This result aligns with the findings reported by Yılmaz et al. (2020) and Babiloni et al. (2017). Furthermore, the reduction of alpha PSD amplitude especially in the posterior regions has been identified as one of the parameters that can discriminate between PDNC and PDCOG (Aarsland et al., 2017). The alpha rhythm prevails during relaxed wakefulness and serves as an indicator of the subject’s attentional capacity and the seamless integration of sensory-motor data, which facilitates the activation of cortico-thalamic and cortico-cortical connections. Consequently, it is unsurprising to observe alterations in this rhythm among patients experiencing cognitive impairment (Mostile et al., 2019).

### 4.3 Diagnostic efficacy and limitations of PDCOG based on QEEG markers

Although significant differences in PAF and PSD characteristics were observed between the two PD groups at the group level, translating these findings into a practical measure for clinical diagnosis in PDCOG remains challenging at this time. Notably, PSD in the alpha frequency range and dominant frequency demonstrated the highest diagnostic accuracy, yet they only achieved moderate AUC values of approximately 0.77. Certain lead measures exhibited remarkably high specificity for PDCOG (reaching up to 93.5% for alpha PSD in T6 with a cutoff below 15.9), indicating that a pronounced shift of PSD towards slower frequencies strongly suggests a diagnosis of PDCOG. However, sensitivity was generally lower, meaning that differentiating between PDCOG and PDNC can be difficult when facing a more typical QEEG pattern. These findings indicate that while changes in PAF and PSD characteristics are specific to cognitive decline, sensitivity is somewhat limited. Therefore, a comprehensive diagnosis should incorporate additional clinical indicators with higher sensitivity.

Compared with previous studies, the diagnostic efficacy of unimodal QEEG in this study (AUC = 0.77) was comparable to multimodal fusion models [e.g., QEEG+MRI combined AUC = 0.77 (Zhang et al., 2021)]. The specificity was significantly higher than that of blood biomarkers [93.5% vs. 77.3% (Liu et al., 2022)]. This difference highlights: The unique advantages of QEEG: low cost, high specificity, suitable for primary care screening; Inherent limitations of a single mode: Heterogeneity in neurodegenerative diseases requires multi-dimensional data complementation.

### 4.4 The link between PAF, alpha PSD and cognition

The modulation of alpha activity by cognitive processes has been well-documented in the literature, suggesting a broad association between alpha activity and various cognitive domains (Klimesch, 2012). Our findings reveal that MoCA scores exhibit a positive correlation with increasing alpha PSD in parieto-occipital leads (P3, O1, O2, T5, T6, PZ), corroborating previous reports (Yılmaz et al., 2020). Furthermore, a study indicates that patients with PD may experience inefficient resource allocation, potentially due to reduced functional inhibition mediated by parietal alpha activity (Weber et al., 2021).

This study unequivocally confirmed the crucial roles of the parieto-occipital region, which has a complex association with PD cognition. An investigation into brain function networks uncovered distinct differences in the parietal and occipital regions between individuals with PD and HC. This discovery implies a possible dysfunction of the parieto-occipital region in PD patients.

Executive dysfunction has been considered the core feature of the cognitive impairment in PD (Arrigoni et al., 2024). Vriend et al. (2015) reported in their study that patients with PD demonstrated compromised performance in comparison to controls while performing a stop-signal task within the inhibition domain. This impairment was accompanied by reduced activation in brain areas linked to inhibitory control. This study discovered a negative correlation between alpha-band PSD and executive function, particularly in specific brain regions such as the right posterior temporal pole, parietal pole, and left occipital pole. This correlation may be attributed to the inactivation of these regions, resulting in a decreased inhibition process (de-inhibition) (van Eimeren et al., 2009). Furthermore, dopaminergic depletion in PD may disrupt the default mode network function, resulting in an inability to properly adjust its activity during executive function tasks. Notably, our research revealed a negative correlation between the executive function score and the alpha-band PSD of the parietotemporal region. This intriguing discovery might be connected to non-disease-specific or compensatory changes in the PD default mode network, ultimately leading to reduced task performance. Interestingly, we found that the executive function score is negatively correlated with the alpha-band PSD of the parietotemporal region. This finding could also be associated with non-disease-specific or compensatory alterations in the PD default mode network, which are linked to diminished task performance.

Moreover, our study revealed a fascinating insight: while PAF has historically been regarded as a reliable measure for evaluating cognitive function, and there is a significant difference in PAF between individuals with PD and healthy controls, this metric is unable to differentiate between PD patients with and without cognitive impairment. This study found a negative correlation between resting-state PAF and the language dimension score of MoCA in PD patients, which may reflect the oscillation-cognition decoupling phenomenon during disease progression. The degeneration of the thalamus-cortex-basal ganglia circuit in PD patients may lead to the dysfunction of *α* rhythm regulation (Dirkx et al., 2017), causing the elevated resting-state PAF (>10 Hz) to lose its cognitive enhancement effect as seen in healthy individuals. Research (Ni et al., 2018) found that basal ganglia neural modulation could significantly alter the power and frequency of the cortical α band, suggesting that dopaminergic drugs may induce oscillation rigidity through a similar pathway, thereby impairing complex cognitive functions. Future studies should combine task-state EEG with multimodal imaging (such as fMRI-PET) to further explore the dynamic relationship between α frequency and the language network at different stages of PD. On the other hand, the PSD index has demonstrated remarkable effectiveness in assessing the cognitive abilities of PD patients, indicating its usefulness in identifying cognitive deficits unique to PD. We aim to explore further the variations in the alpha spectrum and PSD between PD and other types of cognitive impairment, as well as examine the distinct electrophysiological characteristics of cognitive impairment in different diseases.

Our study has certain limitations. First, we utilized the MoCA score, which does not assess specific cognitive domains and may therefore have limited diagnostic accuracy, as a measure of global cognitive function. In our future endeavors, we aim to incorporate more targeted scales for assessment purposes. Second, as an exploratory study, this research aims to preliminarily construct a diagnostic model and identify key features; therefore, cross-validation was not performed. Although this design may limit the direct assessment of the model’s generalizability, the results provide an important foundation for subsequent validation studies. Future work will incorporate larger sample sizes and cross-validation methods to systematically optimize the clinical application potential of the model. Finally, the absence of pathological confirmation in the current study prevents us from establishing the multifactorial pathological mechanism underlying early cognitive decline in PD patients. To address this limitation in future research, we intend to include additional evaluation indicators, such as serological and imaging markers, to explore multimodal markers of PD cognitive impairment.

## 5 Conclusion

In conclusion, the present findings reveal a clear association between alpha PSD and PD cognitive function. These results strongly imply that alpha PSD could be a key factor in evaluating cognitive abilities. Moreover, this study identified the P3α PSD, T5α PSD and T6α PSD as highly promising tools for assessing cognitive function in PD. These indicators may serve as useful auxiliary measures for future assessment.

## Data Availability

The raw data supporting the conclusions of this article will be made available by the authors, without undue reservation.
