# Correction: Peak alpha frequency and alpha power spectral density as vulnerability markers of cognitive impairment in Parkinson's disease: an exploratory EEG study

**DOI:** 10.3389/fnins.2025.1745577

**Published:** 2025-12-03

**Authors:** Yuqing Zhao, Jiayu Cai, Jian Song, Haoran Shi, Weicheng Kong, Xinlei Li, Wei Wei, Xiehua Xue

**Affiliations:** 1 The Affiliated Rehabilitation Hospital, Fujian University of Traditional Chinese Medicine, Fuzhou, China; 2 College of Rehabilitation Medicine, Fujian University of Traditional Chinese Medicine, Fuzhou, China; 3 Fujian Provincial Rehabilitation Industrial Institution, Fujian Provincial Key Laboratory of Rehabilitation Technology, Fujian Key Laboratory of Cognitive Rehabilitation, Fuzhou, China

**Keywords:** cognitive impairment, peak alpha frequency, power spectral density, Parkinson's disease, EEG

In the **Abstract**, the word “positively” was erroneously included in the **Results** section. This sentence has been corrected to read:

PDCOG showed lower alpha PSD in parieto-occipital/posterior temporal regions (P3, P4, O1, T5, T6, PZ) vs. PDNC (*p* < 0.05), with these regions correlating with MoCA scores.

The original version of this article has been updated.

