# Supplementary material for: Peak alpha frequency and alpha power spectral density as vulnerability markers of cognitive impairment in Parkinson’s disease: an exploratory EEG study
Source: Front Neurosci. 2025 Apr 29;19:1575815. doi: 10.3389/fnins.2025.1575815 (PMC12069304; doi:10.3389/fnins.2025.1575815)
Supplement: Supplementary file 1 [file Table_1.docx]

**Supplementary table1**

**clinical characteristics of PDCOG and PDNC**

|  | PDCOG | PDNC | *t*/x² | *P* |
| --- | --- | --- | --- | --- |
| Number of samples | 31 | 13 |  |  |
| Sex(M/F) | 17/14 | 3/10 | 3.73 | 0.054 |
| Age, Y | 67.45±5.71 | 63.38±10.87 | 1.28 | 0.221 |
| Education , Y | 9.74±4.64 | 12.62±3.38 | -2.04 | 0.051 |
| Duration of disease, y | 4.44±3.19 | 4.12±2.72 | 0.32 | 0.753 |
| Hoehn-Yahr | 2.39±0.67 | 2.15±0.56 | 1.11 | 0.274 |
| MDS-UPDRSIII | 32.74±15.31 | 28.85±12.03 | 0.82 | 0.419 |
| MoCA | 20.65±3.48 | 27.85±1.63 | -7.11 | ＜0.001* |

T test: Compared to PD **P*＜0.05.

Whilst categorical variable is presented with number of patients. PDCOG, PD with cognitive impairment; PDNC, PD with normal cognition; MoCa, Montreal Cognitive Assessment; HY, Hoehn and Yahr stage; MDS-UPDRSIII, the partIII of the Movement Disorder Society-Sponsored Revision of the Unified Parkinson's Disease Rating Scale.

**Supplementary table2**

**Comparison of PD and HC in PAF of different brain regions**

| PAF in EEG lobes | | PD | HC | *t* value | *P* | *q* |
| --- | --- | --- | --- | --- | --- | --- |
| Frontal lobe | FP1 | 8.93±0.88 | 9.40±0.74 | -2.43 | 0.017* | 0.023* |
|  | FP2 | 8.93±0.81 | 9.43±0.76 | -2.73 | 0.008* | 0.013* |
|  | F3 | 8.93±0.91 | 9.41±0.75 | -2.48 | 0.116 | 0.116 |
|  | F4 | 9.03±1.11 | 9.42±0.75 | -1.68 | 0.096 | 0.096 |
|  | F7 | 8.95±0.88 | 9.45±0.68 | -2.69 | 0.009* | 0.013* |
|  | F8 | 9.01±1.08 | 9.39±0.75 | -1.73 | 0.087 | 0.087 |
| Temporal lobe | T3 | 9.17±1.18 | 9.65±0.84 | -2.00 | 0.049* | 0.049* |
|  | T4 | 9.04±1.01 | 9.59±0.99 | -2.40 | 0.019* | 0.023* |
|  | T5 | 9.17±1.02 | 9.78±0.87 | -2.74 | 0.008* | 0.013* |
|  | T6 | 9.08±0.92 | 9.67±0.89 | -2.77 | 0.007* | 0.013* |
| Parietal-occipital lobe | P3 | 9.19±1.12 | 9.94±1.05 | -2.95 | 0.004* | 0.013* |
|  | P4 | 9.15±1.04 | 9.96±1.09 | -3.29 | 0.002* | 0.013* |
|  | O1 | 9.03±0.93 | 9.64±0.79 | -2.97 | 0.004* | 0.013* |
|  | O2 | 9.03±0.84 | 9.74±0.97 | -3.41 | 0.001* | 0.013* |
| Central lobe | C3 | 9.10±.122 | 9.88±1.06 | -2.89 | 0.005* | 0.013* |
|  | C4 | 9.14±1.28 | 9.88±1.07 | -2.68 | 0.009* | 0.013* |
|  | FZ | 8.92±0.87 | 9.44±0.75 | -2.75 | 0.007* | 0.013* |
|  | CZ | 9.04±0.98 | 9.65±0.93 | -2.77 | 0.007* | 0.013* |
|  | PZ | 9.15±1.05 | 9.91±1.01 | -3.13 | 0.003* | 0.013* |

T test: Compared to PD **P*＜0.05. FDR-corrected results (Benjamini-Hochberg method, α=0.05). *q*: FDR corrected P value with Benjamini-Hochberg. *q*＜0.05*.

PAF in the frontal, temporal, parietal-occipital and central regions of brain of PD and HC. Except F3, F4 and F8 lobe, the PAF values in various brain regions of PDCOG were significantly lower than those observed in PDNC (*P*＜0.05). PD, patients with parkinson’s disease; HC, health controls; PAF, peak alpha frequency.

**Supplementary table3**

**Comparison of PDCOG and PDNC in PSD of different brain regions**

| PSD in EEG lobes | | PDCOG | PDNC | *t* value | *P* | *q* |
| --- | --- | --- | --- | --- | --- | --- |
| Frontal lobe | FP1 | 8.54±6.48 | 11.60±5.80 | -1.47 | 0.149 | 0.245 |
|  | FP2 | 8.58±6.69 | 11.65±6.94 | -1.37 | 0.177 | 0.252 |
|  | F3 | 10.91±8.97 | 13.86±6.10 | -1.18 | 0.245 | 0.326 |
|  | F4 | 11.65±8.87 | 15.82±9.73 | -1.39 | 0.173 | 0.252 |
|  | F7 | 5.84±4.19 | 7.88±3.87 | -1.51 | 0.139 | 0.245 |
|  | F8 | 5.86±4.35 | 7.32±4.60 | -0.99 | 0.326 | 0.326 |
| Temporal lobe | T3 | 4.26±3.23 | 6.37±4.54 | -1.75 | 0.088 | 0.174 |
|  | T4 | 4.26±3.18 | 5.47±3.04 | -1.16 | 0.252 | 0.326 |
|  | T5 | 7.84±5.51 | 14.29±8.27 | -3.05 | 0.004* | 0.023* |
|  | T6 | 8.58±6.30 | 15.52±8.31 | -3.03 | 0.004* | 0.023* |
| Parietal-occipital lobe | P3 | 10.77±6.47 | 19.81±10.35 | -3.52 | 0.001* | 0.013* |
|  | P4 | 11.96±7.88 | 19.80±10.06 | -2.77 | 0.008* | 0.034* |
|  | O1 | 10.79±7.54 | 19.61±13.00 | -2.83 | 0.007* | 0.030* |
|  | O2 | 11.21±7.87 | 21.84±17.63 | -2.09 | 0.055 | 0.139 |
| Central lobe | C3 | 11.03±7.21 | 15.75±8.77 | -1.86 | 0.070 | 0.139 |
|  | C4 | 12.03±8.36 | 16.11±10.23 | -1.38 | 0.174 | 0.252 |
|  | FZ | 13.25±9.86 | 17.30±9.03 | -1.27 | 0.210 | 0.326 |
|  | CZ | 15.28±9.67 | 20.68±11.92 | -1.58 | 0.122 | 0.245 |
|  | PZ | 15.22±9.92 | 24.93±13.54 | -2.65 | 0.011* | 0.045* |

T test: Compared to PDNC **P*＜0.05. *q*: FDR corrected P value with Benjamini-Hochberg. *q*＜0.05*.

PSD in the frontal, temporal, parietal-occipital and central regions of brain of PDCOG and PDNC. PDCOG, patients with parkinson’s disease; HC, health controls; PAF, peak alpha frequency.

**Supplementary table4**

**Subdivision correlation matrix of MoCA subitem score and PAF**

| correlation |  |  |  |  |  |  |  |  |
| --- | --- | --- | --- | --- | --- | --- | --- | --- |
|  |  |  | MoCA | Memory | Visuospatial | Language | Attention | Executive |
| Spearman Rho | FP1 | r | -0.273 | -0.262 | -0.148 | -0.238 | -0.201 | -0.065 |
|  |  | Sig. | 0.073 | 0.086 | 0.338 | 0.12 | 0.192 | 0.675 |
|  |  | N | 44 | 44 | 44 | 44 | 44 | 44 |
|  | FP2 | r | -0.248 | -0.177 | -0.099 | -0.235 | -0.266 | -0.141 |
|  |  | Sig. | 0.105 | 0.25 | 0.521 | 0.125 | 0.08 | 0.36 |
|  |  | N | 44 | 44 | 44 | 44 | 44 | 44 |
|  | C3 | r | -0.278 | -0.231 | -0.221 | -0.297 | -0.1 | -0.119 |
|  |  | Sig. | 0.068 | 0.131 | 0.149 | 0.05 | 0.519 | 0.44 |
|  |  | N | 44 | 44 | 44 | 44 | 44 | 44 |
|  | C4 | r | -0.221 | -0.15 | -0.224 | -0.276 | -0.125 | -0.066 |
|  |  | Sig. | 0.149 | 0.33 | 0.144 | 0.07 | 0.421 | 0.669 |
|  |  | N | 44 | 44 | 44 | 44 | 44 | 44 |
|  | P3 | r | -0.171 | -0.092 | -0.205 | -0.261 | -0.127 | -0.055 |
|  |  | Sig. | 0.266 | 0.554 | 0.181 | 0.087 | 0.41 | 0.725 |
|  |  | N | 44 | 44 | 44 | 44 | 44 | 44 |
|  | P4 | r | -0.248 | -0.111 | -.321* | -.358* | -0.099 | -0.116 |
|  |  | Sig. | 0.105 | 0.473 | 0.034 | 0.017 | 0.521 | 0.453 |
|  |  | N | 44 | 44 | 44 | 44 | 44 | 44 |
|  | 01 | r | -0.213 | -0.137 | -0.186 | -0.223 | -0.168 | -0.095 |
|  |  | Sig. | 0.164 | 0.376 | 0.227 | 0.146 | 0.277 | 0.54 |
|  |  | N | 44 | 44 | 44 | 44 | 44 | 44 |
|  | 02 | r | -.327* | -0.214 | -0.243 | -.315* | -0.22 | -0.198 |
|  |  | Sig. | 0.03 | 0.163 | 0.112 | 0.037 | 0.152 | 0.197 |
|  |  | N | 44 | 44 | 44 | 44 | 44 | 44 |
|  | F7 | r | -0.248 | -0.251 | -0.162 | -0.228 | -0.214 | -0.024 |
|  |  | Sig. | 0.105 | 0.1 | 0.293 | 0.137 | 0.163 | 0.877 |
|  |  | N | 44 | 44 | 44 | 44 | 44 | 44 |
|  | T4 | r | -0.211 | -0.177 | -0.047 | -0.156 | -0.228 | -0.137 |
|  |  | Sig. | 0.169 | 0.252 | 0.764 | 0.313 | 0.137 | 0.374 |
|  |  | N | 44 | 44 | 44 | 44 | 44 | 44 |
|  | T5 | r | -0.214 | -0.094 | -0.274 | -0.294 | -0.08 | -0.113 |
|  |  | Sig. | 0.164 | 0.544 | 0.072 | 0.053 | 0.604 | 0.467 |
|  |  | N | 44 | 44 | 44 | 44 | 44 | 44 |
|  | T6 | r | -0.159 | -0.046 | -0.226 | -0.223 | -0.108 | -0.1 |
|  |  | Sig. | 0.303 | 0.769 | 0.141 | 0.146 | 0.487 | 0.519 |
|  |  | N | 44 | 44 | 44 | 44 | 44 | 44 |
|  | Fz | r | -0.246 | -0.239 | -0.146 | -0.247 | -0.155 | -0.065 |
|  |  | Sig. | 0.108 | 0.119 | 0.345 | 0.106 | 0.316 | 0.674 |
|  |  | N | 44 | 44 | 44 | 44 | 44 | 44 |
|  | CZ | r | -0.271 | -0.223 | -0.207 | -0.238 | -0.158 | -0.123 |
|  |  | Sig. | 0.075 | 0.146 | 0.177 | 0.12 | 0.305 | 0.425 |
|  |  | N | 44 | 44 | 44 | 44 | 44 | 44 |
|  | PZ | r | -0.265 | -0.122 | -.314* | -.332* | -0.111 | -0.145 |
|  |  | Sig. | 0.083 | 0.429 | 0.038 | 0.028 | 0.473 | 0.347 |
|  |  | N | 44 | 44 | 44 | 44 | 44 | 44 |

r: Correlation coefficient.

**At level 0.01 (two-tailed), the correlation was significant.

*At level 0.05 (two-tailed), the correlation was significant.
